# Curative Treatment of Puerperal Eclampsia

**Published:** 1907-12

**Authors:** Joseph Eve Allen

**Affiliations:** Augusta, Ga.


### Journal-Record of Medicine

Successor to Atlanta Medical and Surgical Journal, Established 1855, and Southern Medical Record, Established 1870.

OWNED BY THE ATLANTA MEDICAL JOURNAL CO.

**Published Monthly.**

*Official Organ Fulton County Medical Society, State Examining Board, Presbyterian Hospital, Etc.*

VOL. IX. DECEMBER, 1907. No. 9

BERNARD WOLFF, M. D., EDITOR. 319-320 Prudential Bldg.

GEORGE BROWN, M. D., BUSINESS MANAGER. Business Office 1½-5½ S. Broad St.

E. G. BALLENGER, M. D., ASSOCIATE EDITOR AND ASSISTANT BUSINESS MANAGER.

### ORIGINAL COMMUNICATIONS.

### CURATIVE TREATMENT OF PUERPERAL ECLAMPSIA

JOSEPH EVE ALLEN, AUGUSTA, GA.

The curative treatment of puerperal eclampsia is one of the most unsettled matters in the whole range of obstetric practice. Upon this subject the widest difference of opinion obtains and men of equal eminence and authority advocate entirely opposite methods of procedure. This confusion is due to the fact that the pathology of this affection has not as yet been fully worked out and still presents a fertile field for future investigation. In order to elicit discussion of this very important subject and get an expression of the views of representative medical men of this section assembled here on this occasion, the following paper is presented embodying my experience in the treatment of puerperal eclampsia based on the observation of cases both in private and hospital practice.

The indications to be met in the treatment of puerperal eclampsia are four in number, viz:

1st. The arrest of the convulsion.

2nd. The removal of all sources of reflex irritation and the sedation of increased nervous irritability.

3rd. The elimination of toxins from the blood.

4th. The sustaining of the patient during convalescence.

These several ends are attained by the administration of drugs combined with certain operative procedures. When a woman, whether she be pregnant, parturient or puerperal, is in the pre-eclamptic state, or actually in convulsions, chloroform should be at once administered by inhalation and this should be continued until the attack is either aborted or has subsided. The pre-eclamptic state indicated by pallor of face, drawing of the head from side to side, fixed stare, twitching of the eyelids and spasm of the respiratory muscles is the time when chloroform exerts its best effect. Often a few whiffs will prevent the coming on of general toxic convulsions and arrest for a while, the seizures. This drug should never be given between the convulsions and must always be sparingly used and with caution. The aim being to give only so much as is necessary to control the fits and no more; for in these cases, chloroform is not devoid of danger, owing to its depressing effect upon the heart and its tendency to produce uterine relaxation and unfortunate results from these causes have been frequently reported as following its use. Ether, as an anaesthetic in eclampsia is altogether contraindicated and under no circumstances should ever be employed because of its irritant action on the lungs and kidneys favoring the occurence of pulmonary edema and increasing the renal insufficiency.

The removal of all sources of reflex irritation is accomplished by giving purgatives, stimulating enemata, and emptying the uterus if the woman be pregnant, or in any of the stages of labor. If the patient is conscious between the spasms or able to swallow, epsom salts in saturated solution is the best purgative to be employed. If, however, she is comatose and swallows with difficulty, or does not swallow at all, croton oil in three drop doses, mixed with butter of glycerine, should be put on the back of her tongue. Elaterium is the ideal purgative for eclamptic cases, but it rapidly deteriorates and hence as sold in the shops is inert and cannot be depended on. Enemata simply to unload the lower bowel should always contain at least one teaspoonful of spirits of turpentine in an emulsion with raw egg or mucilage. As to the best method of terminating the pregnancy and emptying the uterus, the profession is widely divided. The German school of obstetricans advocate the rapid dilatation of the cervix, either by the fingers or steel dilators or multiple incisions and the extraction of the child by version or the forceps. This method of rapid delivery known as the "accouchment force" is condemned by Veit, Charpentier and others who urge less radical means of accomplishing the same result. They induce labor by the use of the bougie or cervical tamponade and leave the first stage of labor to be finished by uterine action. After full dilation is effected, the second stage is expedited by version or the forcep. During the first fifteen years of my practice, the "accouchment force" was always resorted to in every case of eclampsia occurring during pregnancy, or in the first stage of labor, but increased experience has led me to change my methods in this particular, with, I am sure, good results. Some writer has said "treat the convulsions and let the labor take care of itself" and the more I see of eclampsia, the more fully convinced am I that he is right. I believe that it should be the invariable rule in the interest of both the mother and the child, to terminate the pregnancy whenever a woman has had an eclamptic fit, but the means employed should be those that effect this end with the least perturbation and shock to the nervous system. The manipulations incident to the "accouchment force" increase through reflex action the number and severity of the fits and the patient is always left in a state of depression and shock. If labor is induced by the introduction of a bougie and means are used to combat the toxemia and meet the other indications while dilation is going on often delivery will take place unassisted with surprising ease and rapidity. I have yet to see a case in which I would consider Duhrssen's method of dilating the cervix by multiple incision justifiable. The use of powerful branced dilators, such as Bossi's, is often attended with extensive laceration. These are extremely dangerous instruments and are now fortunately going out of use. If rapid dilation is ever necessary, it should be effected by manipulations with the fingers according to the method advised by Edgar, for in this way only can the operator estimate the amount of dilation and avoid doing unnecessary damage. Manual dilation should never be attempted unless the cervix has been obliterated and the resistence is only at the external os.

In puerperal eclampsia there is increased irritability of the nerve centres due to toxemia. To allay this condition of hyperexcitability, morphia given hypodermatically has long been the main dependence of the profession and by some it is used in heroic doses, frequently four or five grains being given in the course of twenty-four hours and seldom less than half a grain at a dose. The very general favor in which this "morphine treatment of eclampsia" is regarded in Great Britain and some parts of America, is due to the teachings of the obstetricans connected with the Rotunda Hospital, the great maternity hospital of Dublin, where it has been the routine practice for years. The clinical experience of some other large maternities has shown that it is possible to conduct a case of eclampsia to a successful termination without the administration of this drug. It is true that morphia will check the convulsions and that it relaxes the blood vessels and thus lowers arterial pressure and also that it temporarily arrests the metabolic processes of the body and in this way diminishes the amount of waste products thrown into the circulation. The use of morphia, however, interferes with secretion and elimination and adds another poison to the already thoroughly poisoned blood and deepens and makes more intense the coma and cerebral congestion always present in this disease. Many a patient, whose death has been attributed to puerperal eclampsia was really the victim of opium poisoning. There are undoubtedly some cases in which morphia may be used to advantage, but in the great majority of cases we have other remedial agents that will do much more good. Hydrate of Chloral and Bromide of Sodium given per rectum after the inhalation of chloroform is stopped, can be used to effect nervous sedation, but these drugs also in many cases can be dispensed with to advantage. Venesection is one of the most powerful means at our disposal for allaying nervous irritation and irritability and in all cases of eclampsia where the woman is plethoric with a strong bounding pulse and high arterial tension, it should always be resorted to. At the risk of being rated another Dr. Sangrado, I say that the general disuse of bloodletting at the present time, especially in this class of cases, I regard as a decided retrograde in practice which might be changed with benefit to our patients. The abstraction of from eight to sixteen ounces of blood has the happy result of bringing down the pulse in eclamptic cases and relieving the cerebral congestion, and taking at once out of the circulation, a certain amount of the toxins. Of course, there are weak, hydremic women whom it would be hazardous to bleed, yet Williams tells us in his work on Obstetrics that he does not hesitate to bleed even such cases. Ordinarily it is well to follow the rule of the distinguished Prof. Barker who said "in eclampsia when in doubt, bleed." To get the best results from venesection, it should always be followed by the use of normal salt solution, either by intravenous transfusion or by hypodermaclysis or high enema. The normal salt solution acts as a diluant to the poison in the blood and as a stimulant especially to the skin and kidneys and profuse diaphoresis and diuresis is the immediate result of its administration. It should always be borne in mind that the introduction of a large quantity of salt solution into the veins is attended with great risk for it sometimes happens that the patient is drowned in this artificial serum or paralysis of the heart from overdistension may occur. The heart has been subjected to great strain by the convulsions and cannot stand much additional strain put upon it, therefore not more than half of a pint of salt solution should be given intravenously at any one time, and not more than four pints should be thus given in twenty-four hours. Hypodermoclysis or the high enema is the best and safest way of using salt solution in the ordinary run of cases in private practice and it should always be given. When given by high enema, the salt solution may be used copiously and often has the same effect, although not so quickly as when put into the vein.

The great value of veratrum viride in eclampsia is not appreciated by a very large part of our profession and its use is condemned by some very eminent obstetricians, yet it is a drug that possesses almost specific virtues in this affection, and the reason that it is not more generally employed is because the preparations used do not represent its full activity. The only preparation of veratrum viride that can be depended upon is that known as Norwood's tincture. It is given hypodermatically in from five to eight drop doses, followed by three to five drops hypodermatically every half hour until the pulse rate falls to 60 per minute. It is said that as long as the pulse remains at 60 per minute, no convulsions will occur. This is denied by Wright and some other of the recent writers, but I have never seen a woman have a convulsion after this action of the drug had been induced. Veratrum viride slows the pulse and lowers intravascular pressure by dilating the capillaries and thus, as it were, bleeds the woman into her own blood vessels. It is also a nerve sedative and by relieving arterial tension, it acts indirectly as a diaphoretic and diuretic. Venesection and veratrum viride must not be employed in the same case. Veratrum is best for hydremic cases and in eclampsia after delivery. Its use should always be carefully watched and strychnia and whiskey given if symptoms of heart failure threaten.

The elimination of the toxins, whatever they may be, is accomplished by the use of the hot pack, or hot air bath. Of these two agents, the hot pack is easiest to apply and most effective. A patient must never be kept in the hot pack longer than half an hour at a time owing to its depressing effect. The cathartics used for their eliminative action should be salines and calomel. No salt of potash should ever be given, either as a cathartic diuretic or sedative, during the course of pregnancy, for fear of the irritating action of the potash on the kidney, causing renal insufficiency. Pathologists are now agreed that in eclampsia, the liver is the organ mainly at fault, therefore calomel is a very important remedy in the preventive and after treatment of eclampsia, owing to its stimulating effect on the liver, as well as its diuretic action when administered in small doses repeated at short intervals.

In properly sustaining the patient after the convulsions have been arrested and the coma relieved, often depends the success of the treatment. At this time, strychnia and some easily assimilable form of iron is usually indicated. The best preparation of iron for convalescents from puerperal eclampsia is Basham's Mixture. This is a solution of the acetate of iron and ammonia and has decided tonic diaphoretic and diuretic value, and should be given in tablespoonful doses every four hours well diluted with water. The diet in convalescense from this disease, should be during the first few days, fluid in character and consist largely of milk. The patient being directed to drink freely of pure water which need not be the commercial Lithia Waters, unless her financial condition allows her to indulge in this luxury. The return to solid food should be carefully made and indigestible articles should be avoided for a long period of time.

Read Nov. 5th, 1907, before the Medical Ass'n of the Tenth Congressional District of Georgia.

